# Mammo-GAN-Assisted Deep Network Training Scheme for Lesion Detection

**DOI:** 10.1007/s10278-025-01771-5

**Published:** 2025-12-16

**Authors:** Juhun Lee, Robert M. Nishikawa

**Affiliations:** 1 Department of Radiology, The University of Pittsburgh, 200 Lothrop Street, Pittsburgh, PA 15237 USA; 2 Department of Industrial Engineering, The University of Pittsburgh, 1025 Benedum Hall, Pittsburgh, PA 15261 USA

**Keywords:** Cycle-GAN, Lesion Remover, Lesion Simulator, Computer aided detection, Generative AI

## Abstract

The performance of lesion detection deep networks is determined by how well the network can learn key features from borderline cases, but finding such cases is challenging. We propose to increase the number of borderline cases for a given network by converting existing training data through applying a Cycle-GAN-based Lesion Simulator (LS) and Lesion Remover (LR). We developed our LS-LR using the developmental portion (80%) of a 10,414 mammography patch dataset from 4789 unique patients (2416 with lesions and 2312 normals). LS creates lesions in normal patches and LR removes lesions from lesion patches, producing harder-to-distinguish cases. Importantly, training LS-LR at lower training epochs (25%, 50%, 75%) can modify the simulation impact. We trained a ResNet18 using a training set for a lesion detection task (baseline). Using our LS-LR at different training epochs, we converted the training samples to be more challenging and retrained the ResNet18 from scratch using the updated set. Our LS-LR at 50%/75% training epochs created the most effective samples for retraining ResNet18, and it achieved superior performance (AUC = 0.901) compared with the baseline (AUC = 0.870; *p* = 0.00005) on the test mammography dataset. External validation confirmed generalizability: for an independent mammogram dataset (134 lesions, 180 normals), AUC improved to 0.866 from 0.839 (*p* = 0.007); for a chest X-ray dataset (826 nodules/masses, 1642 normals), AUC improved to 0.975 from 0.964 (*p* = 0.011). The results showed that our LS-LR model improved lesion detection performance in mammograms and chest X-ray images by transforming existing data into borderline cases.

## Introduction

In the era of deep learning, training an algorithm requires thousands of unique data points in order for the algorithm to learn the feature space well enough to identify or classify the target objects with high accuracy. In a binary classification, the most important samples are those where their feature distributions overlap. The algorithm’s classification power comes from how well the algorithm learns the hyperplane that divides the two distributions.

Previous studies have shown that there exists key data that are essential for training algorithms [1, 2]. However, identifying and curating such key data is difficult. To tackle this issue, various data augmentation techniques, like rotation, translation, flipping, scaling, and kernel filtering (e.g., Gaussian Blur) [3], have been used to help algorithms learn the feature space better. In addition to data augmentation techniques, non-data augmentation methods explore methods of modifying the training hyperparameters to enhance the learning of key features, thereby improving model accuracy. For instance, He et al. [4] employed a “bag of tricks” that included increasing batch size, adopting cosine learning rate decay, implementing knowledge distillation, and introducing image-label mix-up. Recently, researchers have used more modern Generative Adversarial Network (GAN)–based augmentations to improve performance. A few examples include creating synthetic lesions on real images or digital phantoms [4, 5], or inserting real lesions in different locations on normal or abnormal images [6–8]. Although these studies showed an improvement in performance, the augmentations tended to create samples near existing samples. These augmentations were applied equally to all samples, regardless of how important those samples were for training. Since the most important samples for binary classification are located in the overlapping tails of the feature distributions, these traditional augmentations have limited impact on improving the algorithm’s performance. Also, these key samples can be network-specific; samples that are beneficial for one network may not be useful for another network.

In addition, researchers in the medical imaging field are starting to adopt diffusion modeling for medical image synthesis [9–14] for various downstream tasks, e.g., classification [12], detection [9], segmentation [13], denoising [14], and privacy protection [12]. The diffusion modeling process, reconstructing original images by gradually adding (forward process) and removing (reverse process) Gaussian noise via the Markov process, allows models to synthesize high-quality medical images. However, this diffusion process, trained on the process of gradually adding/removing noise, tends to synthesize images that are too smooth (e.g., less or no high-spatial frequency noise) compared to actual images. In addition, diffusion models require more computational resources than GAN [14]. Although diffusion models could learn the sample distribution well and create high-quality medical images, whether the models can synthesize images in the overlapping tails of the feature distributions is unknown.

Thus, there is a need to develop a new augmentation approach to identify key samples, i.e., the samples in the overlapping feature distribution tails for a given network, and create/synthesize those samples directly with clinically feasible quality (e.g., which resemble noise characteristics of real images).

One study [15] showed that a Cycle-GAN [16]-based Lesion Simulator (LS) and Lesion Remover (LR) can change the appearance of a lesion to that of normal tissue and vice versa, and the resulting outcomes (i.e., the normal style of a lesion and the lesion look of normal tissue) can confuse the computer-aided algorithm trained to classify lesions vs. normal tissue, thus diminishing key features that the algorithm used to distinguish lesions and normal tissue, such that the algorithm could not make the right decision. Another study [17] used the LR as a Lesion Highlighter by contrasting the lesion-removed images with their original, which resulted in an improvement in the lesion detection performance of various deep networks. This indicates that the LR can reduce the typical characteristics of lesions (e.g., high pixel intensity compared to background and spiculated margin) such that its result contrasts well with the original.

Although the LS and LR were able to add lesions on normal tissue and remove characteristics that lesions have, GAN-modified samples still held the features from their original classes. The normal style of lesion cases still showed the characteristics of a lesion, and the lesion style of normal tissue still held the features of normal tissue, although very mammographically occult. These occult cases are the difficult or challenging cases, which in fact are the key data (i.e., the data in the overlapping feature distribution tails) for algorithm training, which can be used to improve its performance beyond what a traditional augmentation technique could achieve.

**Fig. 1:**
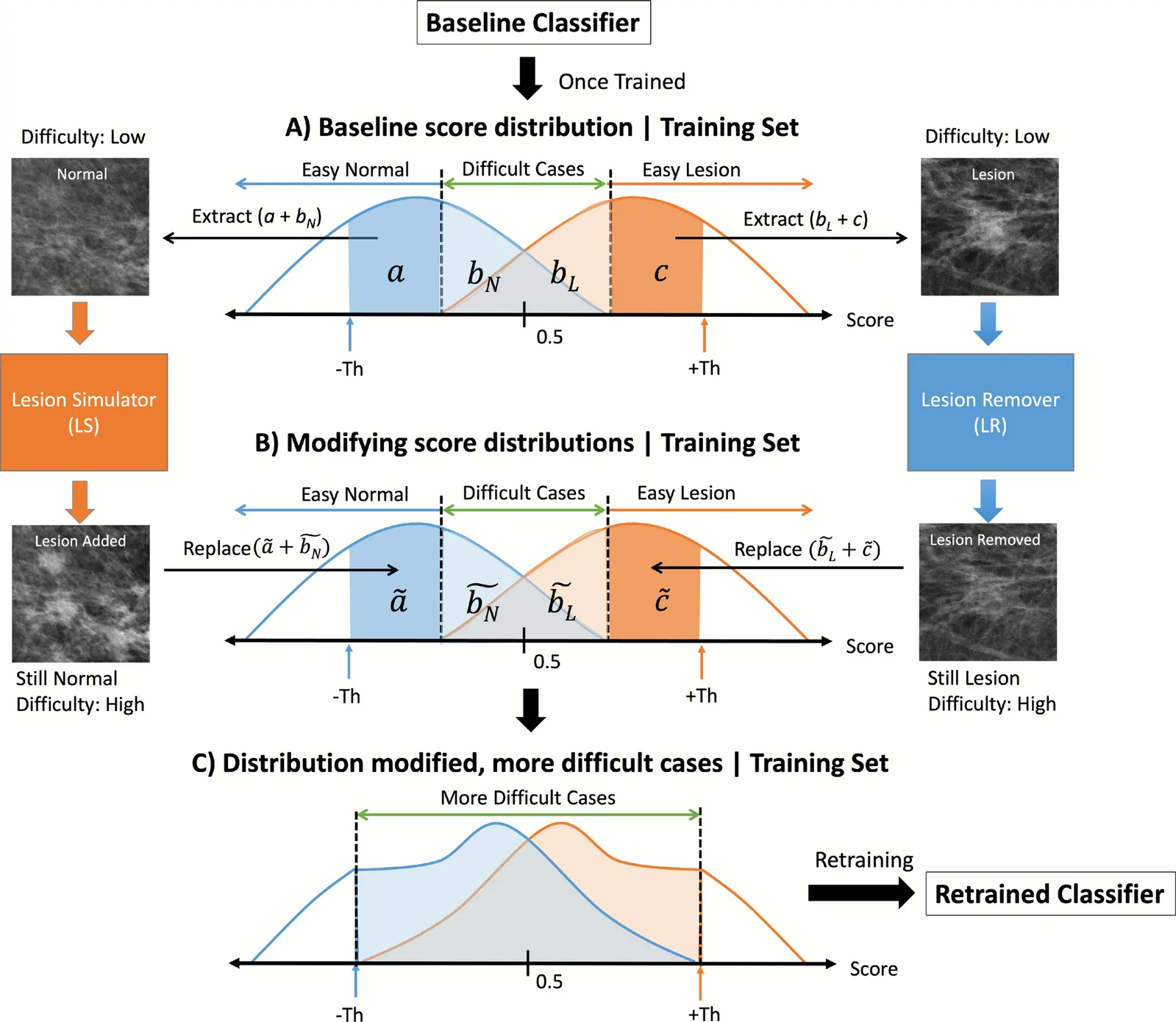
A illustration of our proposed LS-LR model for improving the performance of a lesion detection algorithm.** A** This figure illustrates a hypothetical score distributions obtained by a deep network’s outcome after being trained on the given training set. The distributions will include both easy and difficult patches (lesions and normals) for a given network defined by where the lesion and normal distributions overlap. **B** Our principal idea for using the Lesion Simulator (LS) and Remover (LR) were (i) extracting the portion of easy patches (distribution *a* and *c*) and difficult patches (distribution *b*_N_ and *b*_L_) as shown in **A**, (ii) modifing/converting them using the LS and LR to increase their difficulty level, and (iii) replacing the original samples ($\widetilde{a}$, $\widetilde{{b}_{\mathrm{N}}}$, $\widetilde{{b}_{\mathrm{L}}}$, and $\widetilde{c}$) with the LS and LR modified verions as shown in **B**. **C** By doing the step shown in **B**, we modified the score distributions from **A** to **C**, such that the algorithm could learn key features for better classification by accessing more difficult or challenging patches

Figure 1 illustrates our key idea, increasing challenging samples for a given network by using our LS and LR to convert the existing data in the training set, i.e., the portion of easy lesions (*a* in Fig. 1A) and easy normal tissue (*c* in Fig. 1A), as well as the difficult lesions (*b*_L_ in Fig. 1A) and difficult normal patches (*b*_N_ in Fig. 1A), to be more challenging or occult (Fig. 1C).

We can control the degree of “occultness” by undertraining our LS-LR model. We trained our LS-LR model with 100 epochs, and the resulting networks made the lesions mammographically occult. Some intermediate epochs (e.g., 50 epochs) can provide visually less occult lesions that could serve as key lesion samples to improve the performance of the algorithms. The same scenarios can apply to normal samples.

Our hypothesis is that the training samples modified by the LS-LR model for a given detection network can improve its performance by learning the key features for the task better. In addition, we assumed that our model in intermediate training stages can provide better difficult training samples than those from the final training steps.

In this study, therefore, we tested the possible performance improvements of our LS-LR models for lesion detection tasks. Using ResNet18 [18] as the baseline lesion detector, we tested our LS-LR models for improving lesion detection performance for screening mammograms and then externally validated our models on an independent mammogram patch dataset (134 benign + malignant lesions and 180 normals) and for nodule detection tasks using the Vin Chest X-ray dataset [19].

## Materials and Methods

### Internal Mammography Dataset

Under an approved IRB protocol, we collected screening digital mammograms from 4728 women imaged at a single medical center for routine breast cancer screening from 2017 to 2018. The Selenia Dimension system (Hologic Inc, Marlborough, MA, USA) was used for all mammogram exams. All mammogram sets included the four standard views: left–right cranio-caudal (CC) and left–right medio-oblique (MLO) views. We used the processed views (*for presentation*) for this study.

Among the 4728 women, 2416 had their screening mammograms rated as a recall, BI-RADS classification level 0, with a lesion marked by an MQSA certified radiologist. The remaining 2312 women had screening mammograms with normal readings (BI-RADS 1). Table 1 summarizes the details of the patient information for each group.

**Table 1:** Summary of patient demographic and other information used in this study

	Training		Validation		Testing	
	Normal	Recalled	Normal	Recalled	Normal	Recalled
Sample size (patients)	1264	1150	301	271	465	383
Age						
Mean ± Std	60.9 ± 11.2	59.7 ± 11.9	58.3 ± 10.8	56.3 ± 10.3	60.4 ± 11.2	58.6 ± 11.4
[Min Max]	[24, 93]	[31, 89]	[23, 86]	[37, 85]	[37, 92]	[23, 88]
BI-RADS density						
A	152 (12%)	57 (5%)	35 (11.6%)	7 (2.6%)	52 (11.2%)	10 (2.6%)
B	802 (63.4%)	635 (55.2%)	191 (63.5%)	149 (55%)	307 (66%)	228 (59.5%)
C	300 (23.7%)	444 (38.6%)	68 (22.6%)	111 (41%)	104 (22.4%)	140 (36.6%)
D	10 (0.9%)	14 (1.2%)	7 (2.3%)	4 (1.4%)	2 (0.4%)	5 (1.3%)
Biopsy results						
Non-biopsied	N/A	955 (83%)	N/A	225 (83%)	N/A	318 (83%)
Benign		131 (11.4%)		36 (13.3%)		49 (12.8%)
High risk		14 (1.2%)		3 (1.1%)		4 (1%)
Malignant		50 (4.4%)		7 (2.7%)		12 (3.2%)

**Fig. 2:**
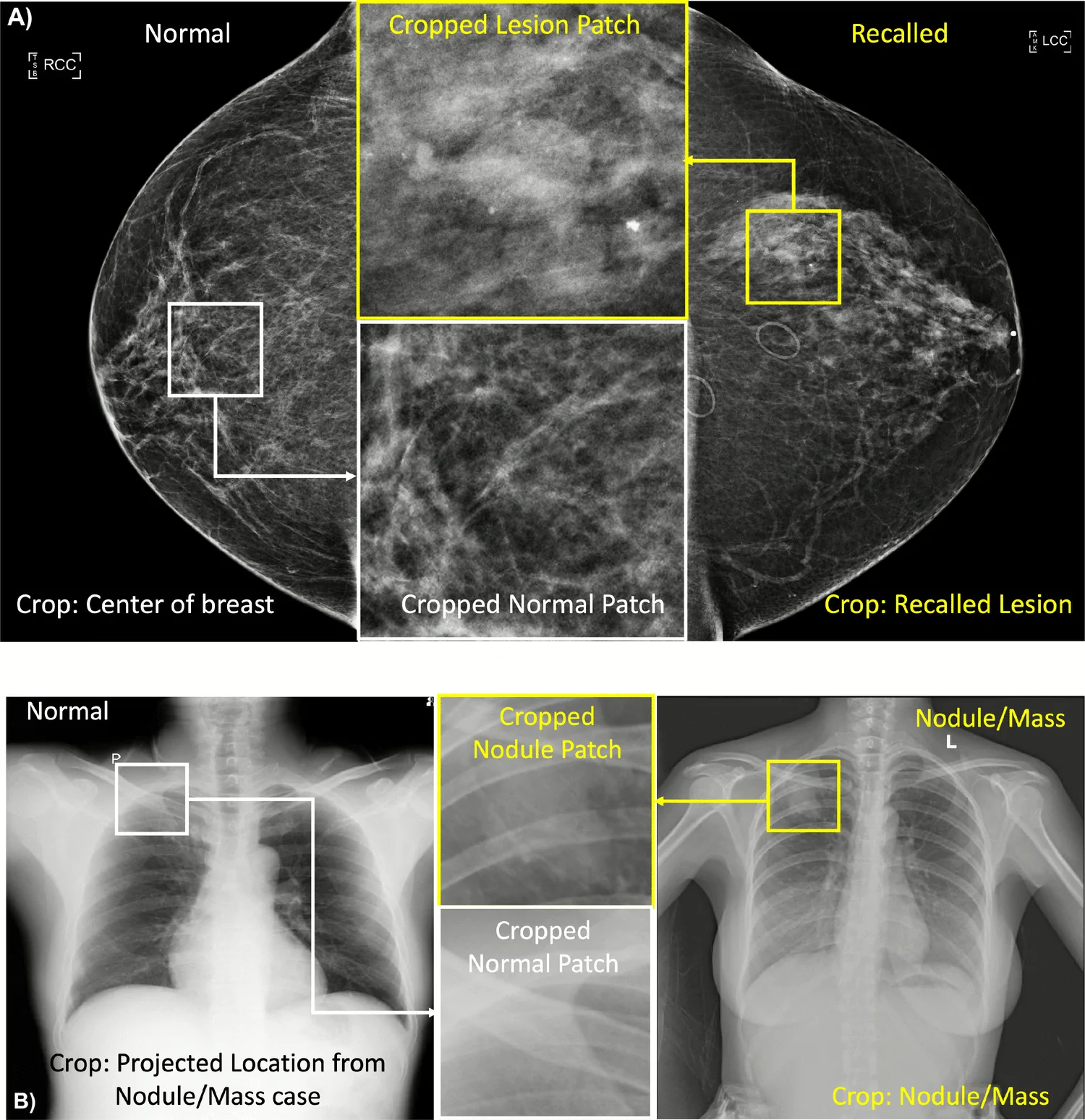
Illustration of how lesion and normal patches were extracted from mammogram and chest X-ray images.** A** This figure illustrates how we extracted 400 by 400 pixel patches from mammograms. For the cases with recalled lesions, we extracted the patch including the lesion. For normal controls, we extracted the centroid of the breast area. **B** This figure illustrates how we extracted 400 by 400 pixel patches from chest X-ray images. For the cases with nodules/masses, we extracted the patch including the nodule/mass. For normal controls, we projected the location of nodule/mass cases to that of normal cases and then extracted a 400 × 400 pixel patch

Using radiologists’ lesion locations, we segmented 400-pixel (2.8-cm) square patches that included the recalled lesions. For controls, we segmented 400-pixel square patches from the breast’s centroid. Figure 2A illustrates this preprocessing.

We then randomly divided the dataset into training, validation, and testing sets with a ratio of 0.64:0.16:0.20 at the patient level. Most recalled lesions were visible on both CC and MLO views, but some were only visible on one view. For developing the LS-LR, we treated lesions from the same patient but in different views as independent samples, while selecting only one lesion (CC) from each patient for testing to avoid correlation between views.

**Fig. 3:**
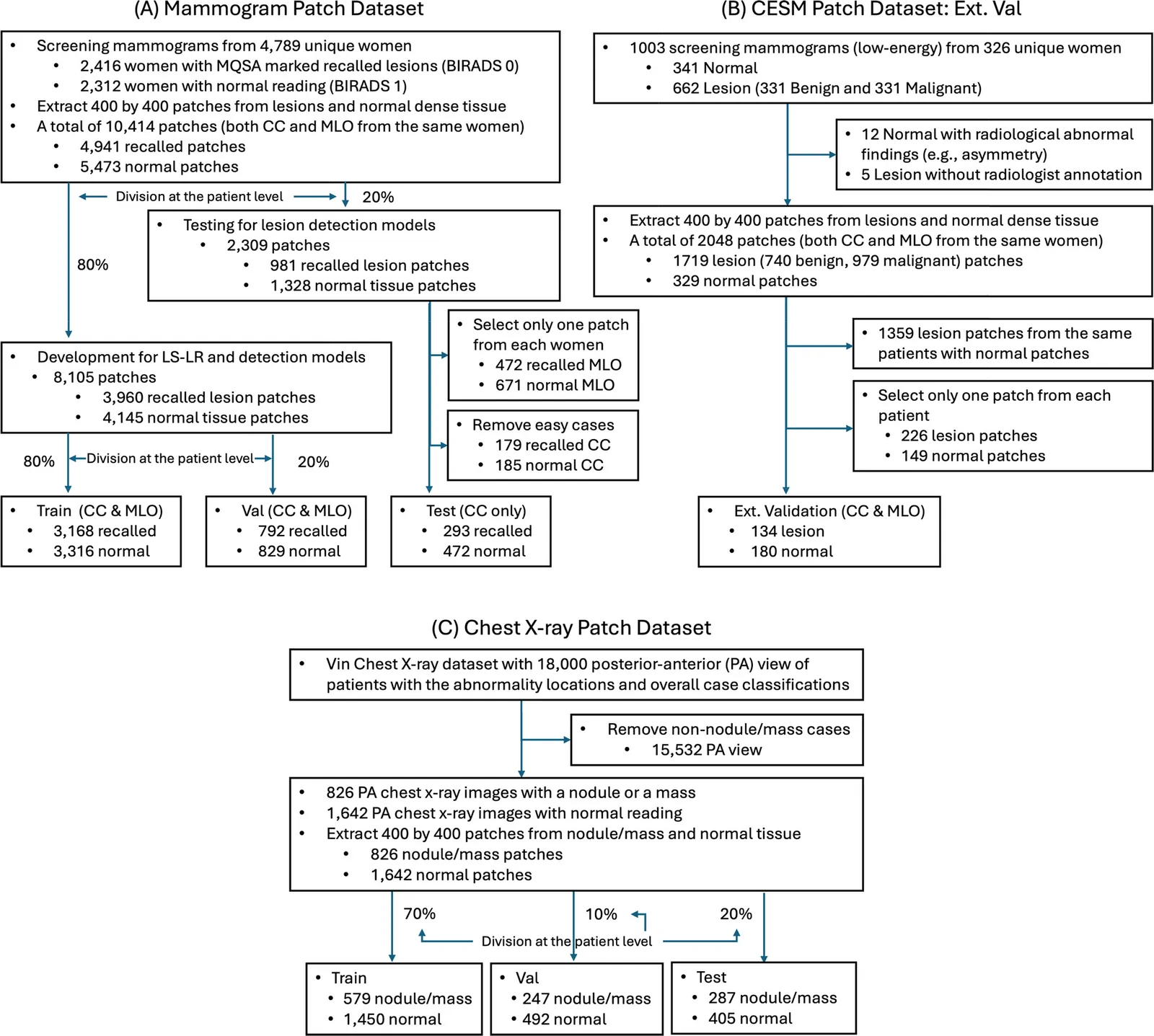
Diagrams of constructing patch data for the study*.* This diagram shows how we curated the training, validation, and testing datasets for our LS-LR models and lesion detection algorithm for our internal mammograms (**A**), external validation mammograms (**B**), and chest X-ray patches (**C**)

In addition, we made the test set more challenging by randomly removing normal cases with a score < 0.02 and lesion cases with a score > 0.98 for the baseline model. As a result, we removed 34% of the easiest test cases and had 8105 patches (3960 recalled) for training and validation combined and 765 patches (293 recalled) for independent testing. Note that the patches in the training, validation, and testing sets were from mutually exclusive patients to prevent information leakage. The detailed diagram of constructing the dataset is given in Fig. 3A. Note that the procedure described above, i.e., intentionally making the dataset more challenging, which is referred to as a “stress test,” is frequently employed in observer studies, for example, by increasing borderline cases or by enriching positive cases beyond the targeted populations (e.g., [20, 21]). Our purpose is to improve the binary classification performance by increasing borderline or challenging cases. That is, we want to show that there is a statistically significant difference between the baseline and LS-LR models; we are less interested in the individual AUC values. The impact of the proposed approach is more evident on a challenging dataset, whereas it may be diminished when evaluated on the full dataset. As identifying the most effective operating points for the LS-LR model requires exploring various parameter combinations, the challenging dataset was used to determine them.

### Exteneral Validation Dataset 1: Mammography

We used the publicly available contrast-enhanced spectral mammography (CESM) dataset [22] as an external testing set on mammography for our LS-LR models. The dataset included 2006 mammography images, consisting of low-energy images, which are the same as screening mammograms, and subtracted images (difference images between low- and high-energy images) of 326 unique women with radiologist-curated lesion locations. The images were obtained using two machines: GE Healthcare Senographe DS (GE HealthCare Technologies Inc., Chicago, IL, USA) and Hologic Selenia Dimensions Mammography Systems (Hologic Inc., Marlborough, MA, USA). Among the 2006 images, 1003 were low-energy mammography images, where 341 were normal, 331 were benign, and 341 were malignant. Note that the pathological ground truth (normal, benign, or malignant) was determined on a per-breast basis; therefore, a single patient could have a malignant finding in one breast and a normal finding in the contralateral breast.

For this study, we used the low-energy mammograms of the normal group as controls and the malignant and benign groups as positive cases. Following the same preprocessing method in the previous section, we extracted a total of 2048 patches with a 400 × 400 pixel size for normal (329) and lesion (1719) samples. Note that there were multiple annotations on the same lesion (up to 10), and therefore, we selected only 1 patch per each lesion. As previously mentioned, the same patient can have both malignant and normal findings. As there were fewer normal cases than lesion cases, we kept the normal patches while removing the lesion patches if they were from the same patient with a normal patch. Lastly, to avoid correlation between views (MLO vs. CC), we randomly selected one lesion per view for each patient for testing. As a result, we curated 180 normal patches and 134 lesion patches for external evaluation. Table 2 summarizes the details of the patient information for each group. The detailed diagram of constructing the dataset is given in Fig. 3B.

**Table 2:** Summary of patient demographic and other information for the external validation mammography set

	Normal	Lesions
Sample size (patches)	180	134
Age		
Mean ± Std	49.6 ± 11.5	51.5 ± 11.8
[Min Max]	[21, 90]	[28, 84]
BI-RADS density		
A	0 (0%)	2 (1.5%)
B	68 (37.8%)	42 (31.3%)
C	102 (56.7%)	80 (59.7%)
D	10 (5.5%)	10 (7.5%)
Biopsy results		
Benign	N/A	74 (55.2%)
Malignant		60 (44.8%)

### Exteneral Validation Dataset 2: Chest X-ray

We used the Vin Chest X-ray dataset [19] as the second external testing set for our LS-LR models. Chest X-rays and mammograms are both X-ray–based techniques, and they share common characteristics, specifically, how lesions appear in the images.

The Vin Chest X-ray dataset is a publicly available dataset containing 18,000 postero-anterior (PA) view chest X-ray scans (15,000 training and 3000 testing) with abnormality locations and case classifications. Among 15,000 images, 826 images had a nodule or mass and were labeled as a positive case. For each of the 826 images, we extracted a 400 × 400-pixel patch centered on the nodule/mass. We then projected the nodule/mass location directly on two different normal images and extracted two normal patches, as shown in Fig. 2B. As a result, we prepared 826 nodule/mass patches and 1642 normal patches. Note that the dataset did not provide demographic and other patient history data for individual patients. Instead, it offered the median age (43.8) and gender breakdown (male:female = 52.2:47.8) for the entire dataset. We then divided the dataset into training:validation:testing sets with a ratio of 7:1:2 at the patient level. The detailed diagram of constructing the dataset is given in Fig. 3C.

### Cycle-GAN-Based Lesion Simulator and Lesion Remover

In this study, we used the original Cycle-GAN architecture from the work of Zhu et al. [16].

A Cycle-GAN consists of two generators, one for the mapping function F:X → Y and another for the mapping function G:Y → X, where X and Y are two different image domains. Briefly, the generator of a Cycle-GAN consists of three sections: an encoder with three convolution layers for compression, followed by a transformer with six residual blocks, and then a decoder with three transposed convolution layers for up-sampling. The discriminator of the Cycle-GAN is called a PatchGAN [23] with a receptive field size of 70 × 70 pixels.

We set the developmental dataset of normal patches as the source domain X and recalled lesion patches as the target domain Y for our Cycle-GAN. Once trained, one generator (*F*) of the Cycle-GAN works as the *Lesion Simulator* (LS) by changing the style of the lesion patch to that of a normal patch. Similarly, the remaining generator (*G*) works as the *Lesion Remover *(LR) by changing the style of the recalled lesion patch to that of a normal tissue patch.

The loss function of the Cycle-GAN included three losses: (1) the adversarial loss to learn the style transfer from one domain to another, (2) the cycle-consistency loss to keep the consistency of the style-transferred images when it transfers back to its original domain (i.e., X → Y and then Y → X), and (3) the identity loss to restrict the transfer within the same domain to be identical (i.e., X → X and Y → Y). There were weight factors *λ*_1_ and *λ*_2_ for the cycle-consistency and the identity loss to regularize their impact for the training.

**Fig. 4:**
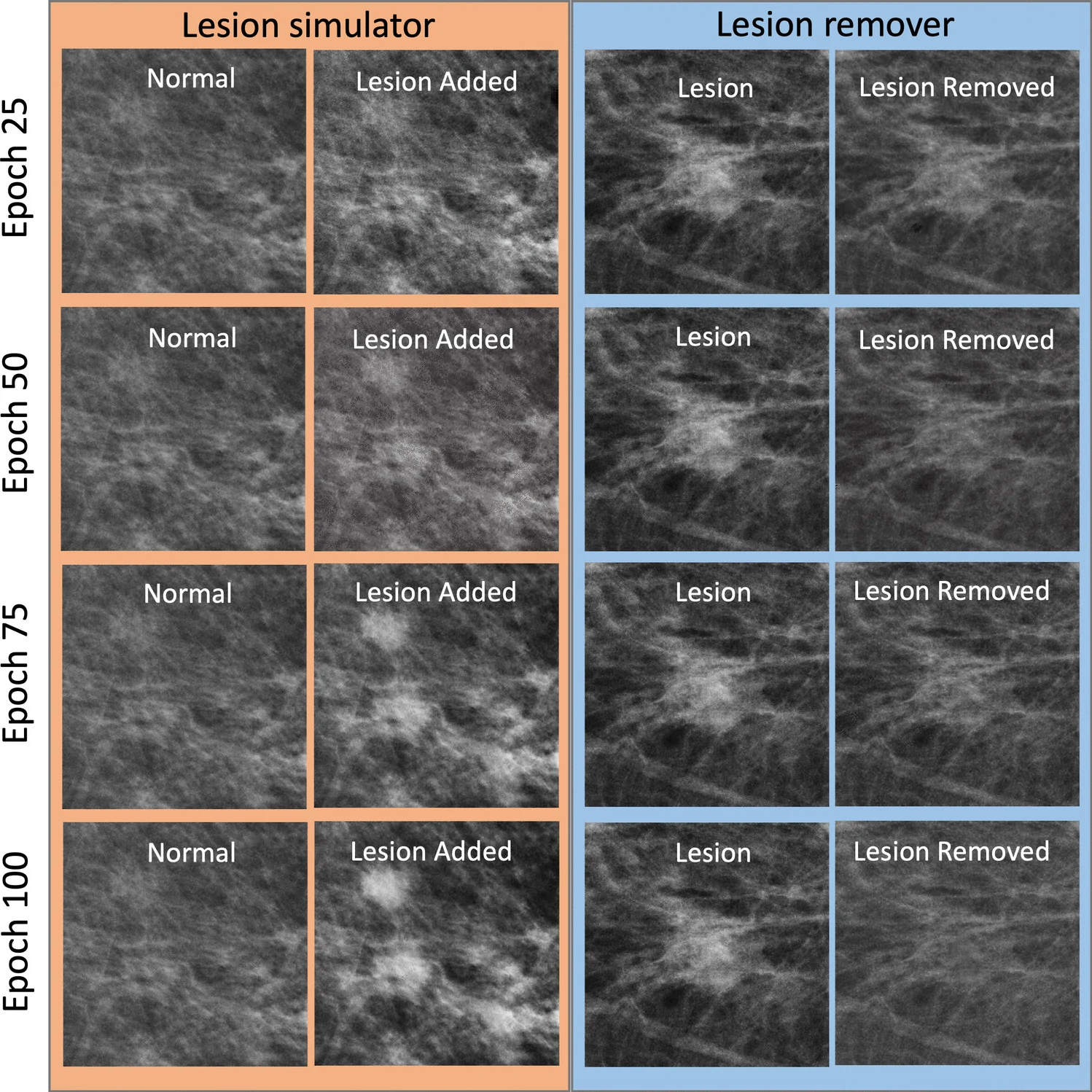
The Lesion Simulator and Lesion Remover outcomes at epochs 25, 50, 75, and 100. Images in the first two columns show the normal tissue patch and its Lesion Simulator (LS) outcome pairs at epochs 25, 50, 75, and 100. Images in the last two columns show the recalled lesion patch and its Lesion Remover (LR) outcomes at epochs 25, 50, 75, and 100. As the epochs increased, the LS added plausible lesions on normal tissue patches, and the LR removed or made the existing lesion subtle. Note that intermediate epochs (25, 50, and 75) showed less apparent impacts by the LS and LR. We postulate that these intermediate results were, in fact, difficult or challenging patches for training lesion detection algorithms, even if the original patches were easy for the given algorithms

We trained the LS-LR model using the mammogram development set. We optimized the Cycle-GAN using an Adam optimizer [24] with a learning rate of 0.0002, and momentum parameters of *β*_1_ = 0.5 and *β*_2_ = 0.999. In addition, we set the maximum epoch as 100 and saved the model at every 5 epochs, and the weights for L1 regularization, *λ*_1_ and *λ*_2_, as 10 and 0.5, and a minibatch size of 4. We used a random left–right vertical flip for data augmentation. We used an Nvidia Titan X GPU with a 12 GB memory for training the networks. Figure 4 shows the LS and LR results for various epochs at 25, 50, 75, and 100 (completion of training). Visually, the LS-LR-modified samples appeared to be difficult or challenging samples for a lesion detection algorithm.

### Creating Challenging Patches for a Detection Algorithm

Our hypothesis is that training samples modified by our LS-LR for a given network will improve the algorithm’s detection performance. In addition, we examined whether the LS-LR in intermediate training steps could provide better challenging patches than those from the final training steps.

As Fig. 4 shows, the LS created pseudo lesions on the normal patches; when used as training samples, these were still labeled as normal. Likewise, the LR removed or made the lesion less conspicuous; these were still labeled as lesion patches. Thus, lesions-added normal patches and lesion-removed lesion patches were challenging normal patches and lesion patches.

To use LS-LR for improving lesion detection algorithms, one needs to decide how many easy samples should be changed by the threshold (*Th*) level (as shown in Fig. 1) and which epoch of LS-LR should be used. To do so, we considered the following *Th* levels of [0.05, 0.1, 0.25, 0.5, 0.75, and 1], and for LS-LR epochs, we evaluated the cases of 25, 50, 75, and 100 epochs.

### Detection Algorithm

To test our hypothesis, we employed ResNet18 [18], which was pretrained on ImageNet [25]. Note that there exist more modern and advanced networks, such as Vision Transformer (ViT), than ResNet18. As our purpose was to show the potential of our LS and LR for improving lesion detection algorithms, the choice of classifier was less important, as long as the model was suitable for fast prototyping. We updated the last few layers of each ImageNet pretrained ResNet18 to match our purpose: to classify the patch as a recalled lesion or normal for mammograms (benign and malignant lesions vs. normal for external validation mammograms) and nodule/mass or normal for chest X-ray images.

We first trained a *baseline* ResNet18 on the training set. Then, we identified the target patches for the LS-LR process in the training set by applying *Th* levels and transformed them by using the LS and LR at certain epoch levels.

For each testing scenario, which is the combination of 6 *Th* levels (i.e., *Th* = [0.05, 0.1, 0.25, 0.5, 0.75, and 1]) and 4 epochs (i.e., LS-LR at epochs of 25, 50, 75, and 100), we retrained the *baseline* ResNet18 using the updated training set. As a result, we developed a total of 24 retrained ResNet18s under different *Th* and epoch combinations to find out which *Th* and epoch for the LS and LR would be most effective.

For training and retraining ResNet18s, we randomly segmented 224 by 224 patches from the original patch images with 400 by 400 pixels. In addition, we employed random vertical and horizontal flips, random rotations in the range of ± 30°, and random scales with a ± 25% range. Using Matlab (MathWorks, Natick, MA USA) as the training environment, we employed the Adam optimizer [24] with an initial learning rate of 0.001, a learning rate dropping factor of 0.1 for every 10 epochs, and momentum parameters of *β*_1_ = 0.5, *β*_2_ = 0.999. In addition, we set the maximum epoch at 50 and a minibatch size of 32. We also employed early stopping when the validation accuracy at each epoch dropped more than 5 times. We used an Nvidia Titan X GPU with a 12 GB memory for training all networks.

### Evaluation Methods

We used the area under the ROC curve (AUC) as our evaluation metric for classifying a given patch with a lesion or not for mammograms, and a nodule/mass or not for chest X-rays. To identify the effective operating points for our LS-LR and compare the correlated AUCs of the *retrained* ResNet18s over the *baseline*, we employed Delong’s method using a two-tailed test [26] with adjusted *p*-values using the false discovery rate (FDR) correction [27] for multiple comparisons. After that, we externally validated our LS-LR models with the selected operating points for the two external validation sets, one mammography and one chest X-ray, by comparing the correlated AUCs of the *retrained* ResNet18s over the *baseline* by using the two-tailed Delong’s method.

## Results

### Selecting Effective Operating Points for Lesion Simulator and Lesion Remover

**Fig. 5:**
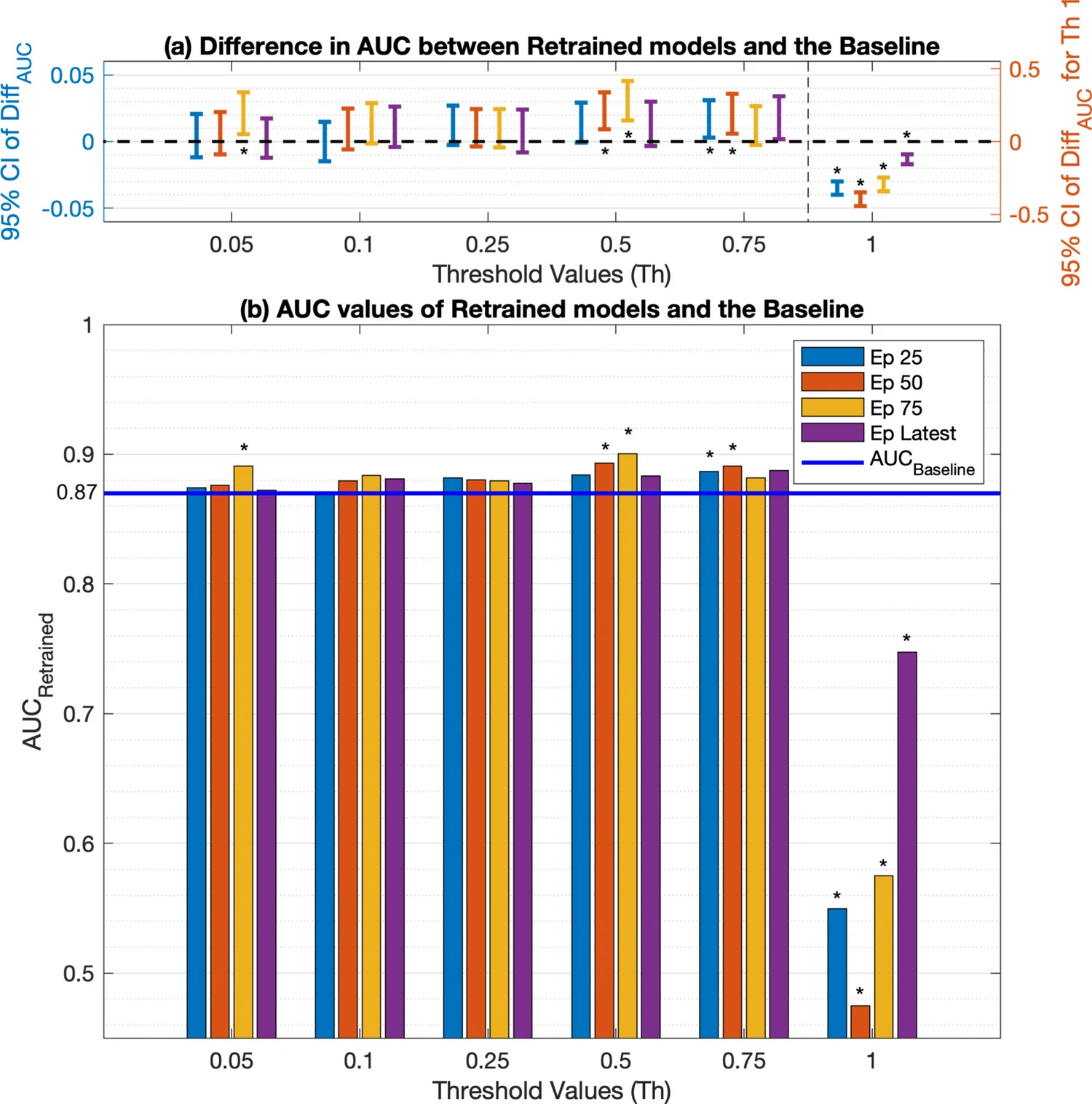
AUCs for Retrained ResNet18 using the LS-LR-modified samples for various training epochs and threshold values. **b** The AUC values of retrained ResNet18 models on the training samples modified by our LS-LR at various training epochs (*Ep*) and with different threshold (*Th*) levels to identify appropriate operating points for our LS-LR process. **a** The 95% confidence intervals (95% CI) and * indicates the statistically significant differences in the AUCs between each model and the baseline after applying the false discovery rate (FDR) correction method. Compared to the AUC of the baseline ResNet18 with an AUC of 0.870, most cases, except *Th* = 1, showed higher AUC values, but only a few showed statistically significant improvement. Among all *Ep* and *Th* combinations, *Ep*50 and *Ep*75 at *Th* = 0.5 showed the best improvements (AUCs = 0.893, 0.901) over the baseline (*p* = 0.001, 0.00005) after the FDR correction. Modifying the entire samples, i.e., *Th* = 1, confused the algorithm and resulted in performance degradation regardless of *Ep*

Figure 5a and b summarizes the AUC values of the *retrained* ResNet18 on the training samples converted by our LS-LR at 4 different training epochs and at 6 different threshold levels on the test portion of our developmental dataset. Compared to the *baseline* (AUC = 0.870 with a 95% confidence interval (95% CI) = [0.841, 0.898]), most cases, except *Th* = 1, showed higher AUC values, but only a few showed statistical significance. These results prove our hypothesis that our training approach using the LS-LR could improve the performance of lesion detection algorithms by converting the existing patches to be more difficult or challenging.

Regarding the best operating *Ep*, we found that *Ep*50 at *Th* = 0.5 (AUC = 0.893, 95% CI = [0.867, 0.919]), *Ep*75 at *Th* = 0.5 (AUC = 0.901, 95% CI = [0.876, 0.926]), and *Ep*50 at *Th* = 0.75 (AUC = 0.891, 95% CI = [0.868, 0.917]) showed statistically significant improvements (95% CI of the difference in AUCs (Diff_AUC_) = [0.009, 0.037], *p*-values = 0.001 for *Ep*50 at *Th* = 0.5, 95% CI of Diff_AUC_ = [0.012, 0.046], *p*-values = 0.00005 for *Ep*75 at *Th* = 0.5, and 95% CI of Diff_AUC_ = [0.006, 0.036], *p*-values = 0.006 for *Ep*75 at *Th* = 0.5) over the *baseline* compared to other epochs, which means they could be the effective operating points for our LS-LR.

Regarding the best operating *Th*, we found that the *Th* at 0.5 and *Th* at 0.75 could be the effective value for operating the LS-LR. Especially, we found that modifying the entire samples, i.e., *Th* = 1, was not effective, as it totally confused the *retrained* ResNet18s. This indicates that some easy or obvious lesions and normals, which are located at the two ends of the score distribution (non-shaded area in Fig. 1), are necessary to train the algorithm properly.

### External Validation of Lesion Simulator and Lesion Remover for Mammograms

**Fig. 6:**
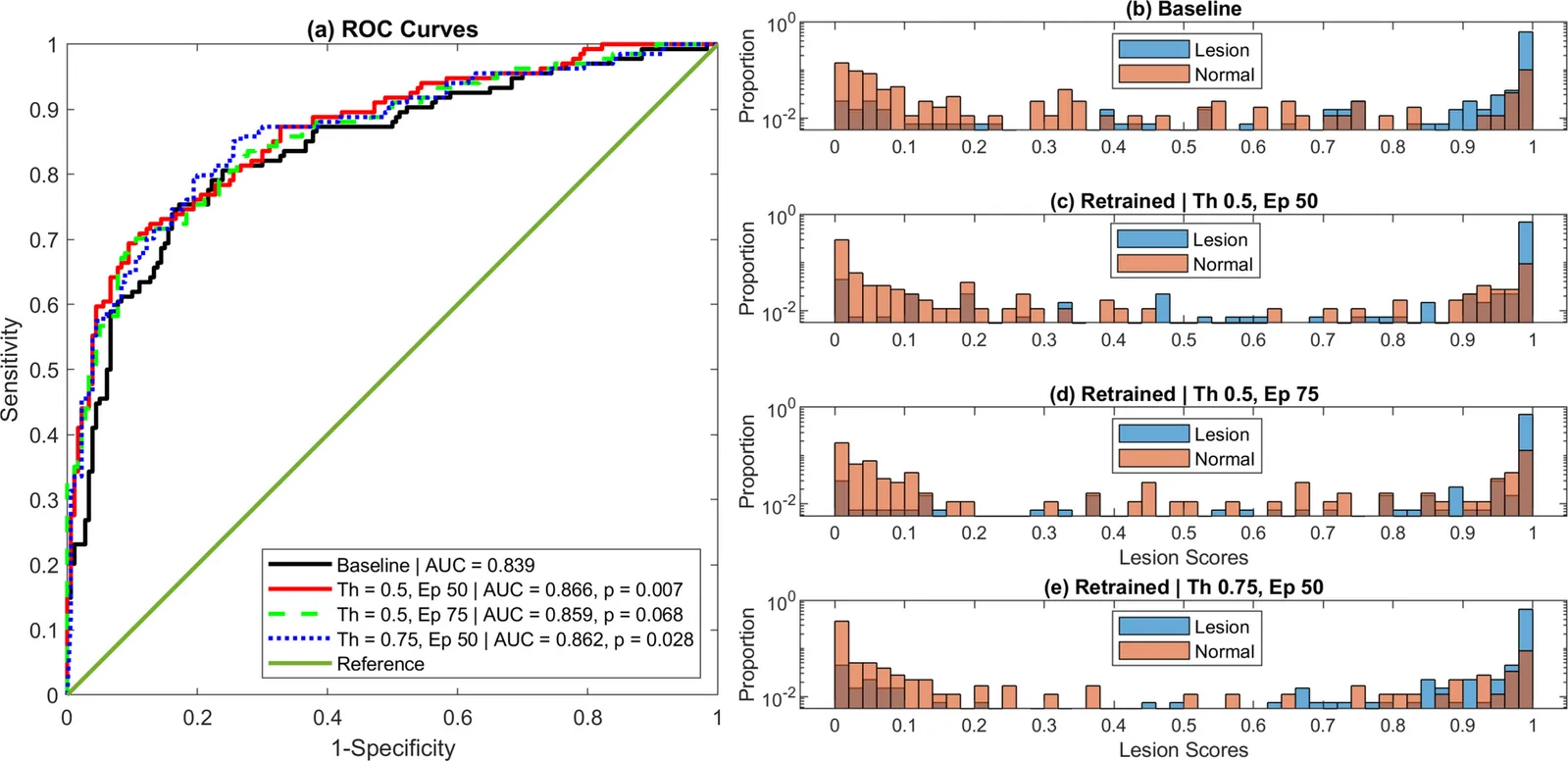
ROC curves and lesion probability distributions for the *baseline* and the *retrained* ResNet18s at *Th* = 0.5 with *Ep*50 or 75 and *Th* = 0.75 with *Ep*50 for lesion detection in the external validation mammograms. Among all combinations of *Ep*s and *Th* levels, *Ep*s 50 and 75 at a *Th* = 0.5 and *Eps* 50 at a *Th* = 0.75 showed an improvement over the baseline (AUC = 0.839). The AUCs of the retrained versions were 0.866, 0.859, and 0.862, and two of them (*Ep*50 at *Th* = 0.5 and 0.75) were statistically better (*p* = 0.007, 0.028) than the baseline. One can observe the increased sensitivity and specificity at the middle operating points in the ROC curves (**a**), shows the improved classification between lesions and normal tissue. In addition, we also observed the increased class separations between lesion and normal patches for the retrained versions (**c**–**e**) over the baseline (**b**) from their score distributions. In fact, the proportions at the two end points (0–0.1 and 0.9–1) increased, while there was less overlap in the middle (score 0.2–0.8). This directly showed the improvement in the lesion score of the retrained versions over the baseline

We validated three of our LS-LR models (*Th* = 0.5 with an *Ep* = 50 or 75, and *Th* = 0.75 with an *Ep* = 50) using patches extracted from an external validation mammography dataset. Figure 6a–e shows the ROC curves and lesion probability score distributions of the *baseline* and *retrained* ResNet18s with training *Eps* 50 and 75 at *Th* = 0.5 and *Eps* 50 at *Th* = 0.75. The baseline AUC was 0.839 with a 95% CI = [0.793, 0.886], which was lower than its performance on the test portion of the development set. This result is expected, given that the external validation dataset was collected at a different site and includes images acquired from a different vendor.

We found that all three operating points (*Eps* 50 and 75 at *Th* = 0.5 and *Eps* 50 at *Th* = 0.75) showed improvement over the baseline (*retrained* AUCs = 0.866, 0.859, 0.862 vs. *baseline* AUC = 0.839). However, only *Ep*50 at *Th* = 0.5 (AUC = 0.866, 95% CI = [0.824, 0.909]) and *Ep*50 at *Th* = 0.75 (AUC = 0.862, 95% CI = [0.819, 0.906]) showed statistically significant improvements (95% CI of the difference in AUCs (Diff_AUC_) = [0.007, 0.047], *p*-values = 0.007 for *Ep*50 at *Th* = 0.5 and 95% CI of Diff_AUC_ = [0.003, 0.044], *p*-values = 0.028 for *Ep*50 at *Th* = 0.75) over the *baseline.*

The ROC curves revealed an overall improvement over the baseline for all operating points. The score distributions showed better class separation, with higher proportions at the ends (0–0.1 for normal and 0.9–1 for lesions) and lower proportions in the middle ranges (0.2–0.8) compared to the *baseline*. These results demonstrate the effectiveness of our LS-LR models in learning key features better.

### External Validation of the Lesion Simulator and Lesion Remover on Chest X-ray Images

We validated three of our mammography-trained LS-LR models (*Th* = 0.5 with an *Ep* = 50 or 75, and *Th* = 0.75 with an *Ep* = 50) using patches extracted from chest X-rays. Note that our LS-LR models were solely based on mammograms, but chest X-rays and mammograms share common characteristics, specifically, how lesions appear in the image. Therefore, we applied our mammogram-based LS-LR models directly on chest X-ray lesion/nodules.

The *baseline* ResNet18 achieved an AUC = 0.964 with a 95% CI = [0.945, 0.985]. The retrained ResNet with LS-LR models achieved an AUC = 0.974 with a 95% CI = [0.957, 0.992] (*Ep* = 50, *Th* = 0.5), AUC = 0.975 with 95% CI = [0.959, 0.992] (*Ep* = 75, *Th* = 0.5), and AUC = 0.974 with 95% CI = [0.958, 0.992] (*Ep* = 50, *Th* = 0.75). These were statistically significantly better than the *baseline* (95% CI of Diff_AUC_ = [0.002, 0.018] and *p*-value = 0.019 for *Ep50 at Th* = 0.5, 95% CI of Diff_AUC_ = [0.002, 0.019] and *p*-value = 0.011 for *Ep75 at Th* = 0.5, and 95% CI of Diff_AUC_ = [0.001, 0.019] and *p*-value = 0.031 for *Ep*50 at *Th* = 0.75).

**Fig. 7:**
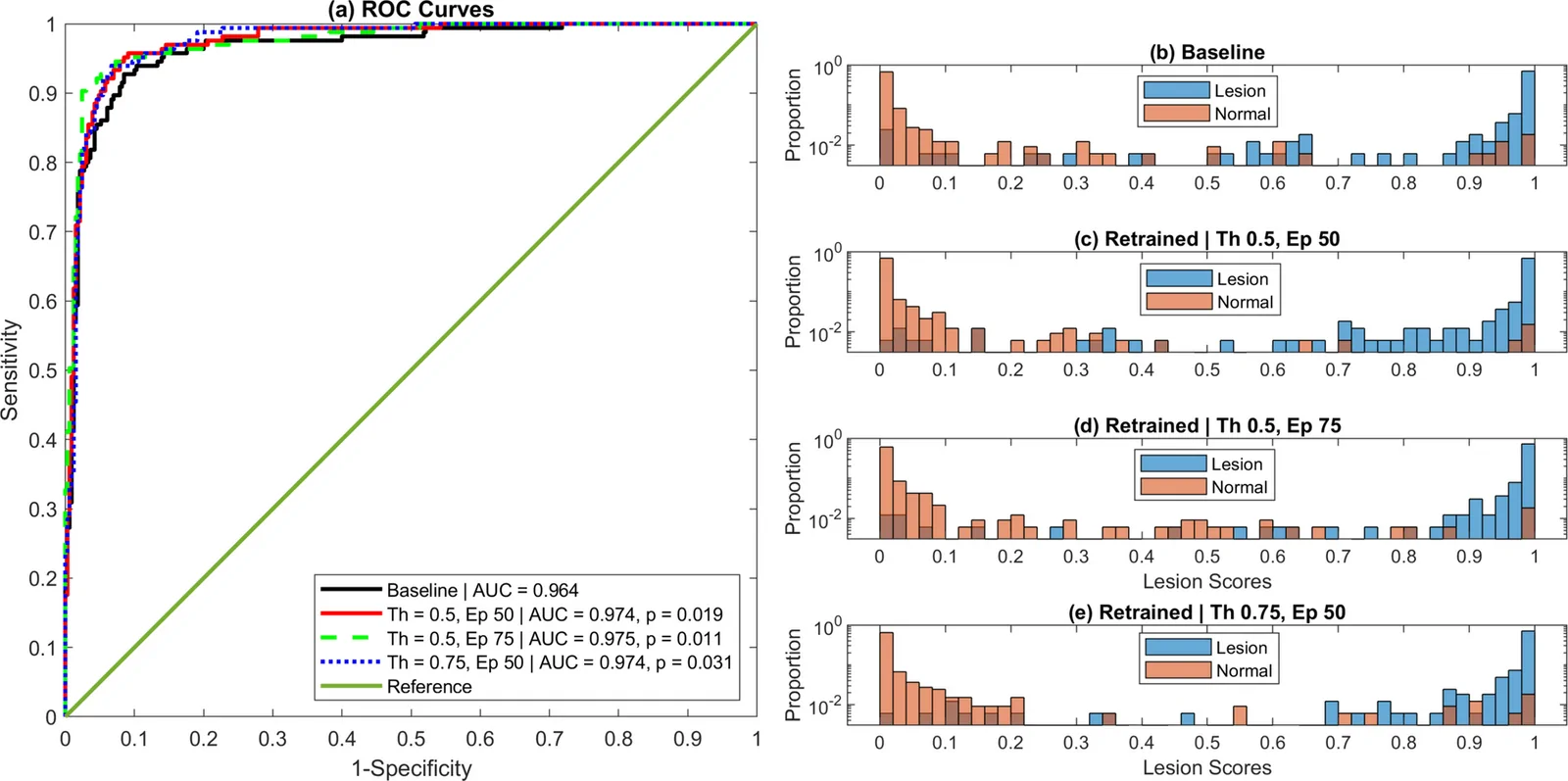
ROC curves and lesion probability distributions for the baseline and the retrained ResNet18 at* Th* = 0.5 with *Ep*50 or 75 and *Th* = 0.75 with *Ep*50 for nodule/mass detection in chest X-ray images. The retrained ResNet18 with *Th* = 0.5 with *Ep*50 or 75 and *Th* = 0.75 with *Ep*50 showed improved AUCs (AUCs = 0.974, 0.975, and 0.974) over the baseline (AUC = 0.964) and were statistically significant (*p* = 0.019, 0.011, and 0.031). One can observe the increased sensitivity and specificity in the middle portion of the operating points in the ROC curves (**a**). In addition, we also observed the increased class separations between nodule/mass and normal patches for the retrained versions (**c**–**e**) over the baseline (**b**). In fact, the proportions at the two end points (0–0.1 and 0.9–1) increased, while there was less overlap (in fact less samples) in the middle (score 0.1–0.9). This directly showed the improvement in the lesion score of the retrained versions over the baseline

Similar to mammogram results, the ROC curves and score distributions showed improved performance over the *baseline* (Fig. 7a). Specifically, we found more samples at the score ends: 0–0.1 for normal and 0.9–1 for nodules/masses. The middle range (0.2–0.8) had less overlap and samples (Fig. 7b–e). These show that our mammography-based LS-LR models are not only effective on the mammography images, but also on the external, independent, and non-mammography images, i.e., the chest X-rays.

## Discussion

In this study, we showed that our proposed models improved the performance of a deep network classifier in identifying positive and negative mammographic patches and lung nodules in chest X-ray patches. The critical aspect of our approach was to create more training data specifically designed to provide more samples near the hyperplane between positive and negative patches for a given deep network. As a result, the network was able to fine-tune its decision hyperplane better than its original (i.e., a network without using our LS-LR models), such that its detection performance improved.

GAN has been used as part of an augmentation tool to increase training samples, especially positive samples (e.g., cancer), for developing deep networks for various tasks (e.g., detection, classification) [3, 28] in the field of medical imaging. Note that the main objective of these previous approaches and that of our LS-LR are similar: to increase important or essential data for the algorithms to learn key features better. However, our LS-LR is unique in three ways. First, we tried to focus on the decision hyperplane between positive and negative samples and increased the samples near it by converting existing samples to be more challenging using our approach, while others created samples to fill the whole feature space. Second, as we converted existing samples, there were no changes in the number of samples for training, while previous studies tended to create more synthetic or simulated images than the given dataset [4], as it is relatively easy to generate them. Although more synthetic data could improve the algorithm’s performance, it is not computationally efficient, as more data should be processed by the algorithm during the training. Third, we converted positive and negative samples equally, while others focused more on increasing the positive samples. Positive cases in medicine are rare, so it makes sense to create more positive samples than negative ones to make the dataset class-balanced. However, negative cases, especially challenging ones, are also important as they, along with the challenging positive cases, are a portion of the key data to determine the hyperplane that the algorithm should identify during the training. In this respect, our LS-LR has a clear advantage over the other methods, as we equally augment challenging negative and positive cases.

Note that we based our LS-LR model on the original Cycle-GAN, which some may consider outdated. However, the choice of a backbone network to create LS-LR is less important, as our purpose was to show a new method to increase difficult or challenging cases for a given network. In fact, a recent study showed that GAN still performed closely to diffusion models [9], and diffusion requires more resources than GAN. Nonetheless, there exists a diffusion model counterpart of the Cycle-GAN, e.g., CycleDiffusion [29], which we can investigate for its possible improvement over the current Cycle-GAN as a future study.

Moreover, it is important to note that we designed our LS-LR to identify network-specific challenging cases after network optimization. Our premise was not on developing an augmentation technique that outperforms existing augmentation techniques. Instead, our LS-LR can be integrated with existing augmentation frameworks, such as image-based augmentation (e.g., random flip) and hyperparameter augmentation (e.g., bag of tricks [30]).

While the difference in AUCs between *baseline* and *retrained* ones with our LS-LR was relatively small, it was statistically significant. The difference also has clinical significance because cases that are incorrectly classified by the model are highly likely to be cases that radiologists find difficult. Small improvements in correctly classifying radiologist-difficult cases could have a larger clinical impact than improving the classification of cases that radiologists can classify correctly without any computer aid.

Further, while correctly detecting breast cancer in a mammogram is the primary goal of screening mammography, identifying challenging negative cases as negative is also important. The number of annual screening mammograms performed in the USA is nearly 42 million [31], with approximately 11% of them receiving a false positive detection [32]. So even a small reduction in the false positive rate could result in tens of thousands of normal patients not being subjected to additional imaging and possibly ending up with an unnecessary biopsy. Therefore, reducing the false positive rate is also critical [33] and having more challenging negative cases for training algorithms may be beneficial.

Lastly, investigators are currently evaluating breast cancer detection algorithms for triaging screening mammograms (e.g., [34, 35]) to identify a subset of screening cases as having a very low probability of containing a cancer that does not need to be read by a radiologist. This could help alleviate the problem of a shortage of breast radiologists. For this AI-based triaging approach to be successful, the false negative rate needs to be extremely low, since a cancer triaged out will not be detected. Therefore, small incremental increases in specificity at high sensitivity become important clinically for triaging applications and can have a clinically significant impact.

We conducted an adhoc analysis on the two tails of ROC curves (see Fig. 7a) to check our method’s potential of improving performance at high specificity/sensitivity levels, by using the partial AUC over the sensitivity level at 0.9 or higher (pAUC_Sens>0.9_) and that over the specificity level at 0.9 or higher (pAUC_Spec>0.9_) as evaluation metrics. We found that, compared to the baseline model, the retrained ResNet18 for chest X-ray using our LS-LR model at *Ep*75 and *Th* = 0.5 had statistically significantly higher pAUC_Sens>0.9_ (0.0827 vs. 0.0771) and higher pAUC_Spec>0.9_ (0.0847 vs. 0.0775) (*p*-values = 0.048 and 0.007, respectively, computed via bootstrap sampling). This result shows that our LS-LR model does not only improve the overall performance but also the two tails of the ROC curves, which is crucial for reducing the number of false detections and when triaging screening cases.

Our study has limitations. First, we used ResNet18 as a baseline lesion detector to show the effectiveness of our LS-LR models. Other modern network architectures (e.g., Vision Transformer [36]) may yield different results. However, our approach identifies target samples customized for the given architecture and converts them to be more challenging, so we expect similar outcomes for other architectures, though their impact may vary. We will investigate our retraining approach with LS-LR models for other modern architectures in the future.

Second, our LS-LR models used a patch-based Cycle-GAN with a 400 by 400 pixel input and output size. We showed it works for patch-based lesion detection, but it is not validated for an entire image. We can apply our LS-LR models to the entire image using a sliding window approach or develop it using the entire image, as done in previous studies [37–39]. Additionally, we can investigate more modern generative networks, like diffusion models (e.g., [40, 41]), to improve generated image quality.

Third, we did not exhaustively search for effective operating points using the LS-LR models. We investigated four training epochs (*Ep* 25, 50, 75, and 100) and six score threshold values (*Th* 0.05, 0.1, 0.25, 0.5, 0.75, and 1), but they do not cover all possible combinations. We found that 50 epochs and thresholds 0.5 and 0.75 could be effective, but other epochs may be better. Effective epochs and/or threshold values for the LS may not be the same for the LR, and vice versa. We prepared the LS-LR at every 5 epochs, so we can investigate finer levels and separate operating points for the LS and LR in a future study.

Lastly, although we externally validated our model using mammography datasets from different hospitals and imaging vendors, additional validation on larger datasets and from other vendors (e.g., Siemens) may be necessary to further assess its generalizability. However, our second external dataset was from a different country (Vietnam), with a different modality (chest X-ray), and a different clinical task (lung nodule detection). Using our mammography-trained network, we were able to generate challenging training cases from the existing chest radiographs that were able to improve the detection of lung nodules. This is a strong stress test of the method and shows the robustness and generalizability of our model.

## Conclusion

In this study, we proposed a training scheme that leverages a Cycle-GAN-based Lesion Simulator (LS) and Lesion Remover (LR) model to enhance the lesion detection performance of deep neural networks. Our LS-LR model can convert existing training data to be more challenging, such that they could serve as borderline cases for a given network. We demonstrated the potential of our approach by showing that a network incorporating our LS-LR model can improve lesion detection performance in both mammograms and chest X-ray images.

## Data Availability

All data needed to evaluate the conclusions in the paper are present in the paper. The datasets such as original screening mammography images and their related BI-RADS assessments used and/or analyzed during the current study are available from the corresponding author on reasonable request. The Vin Chest X-ray dataset is a publicly available dataset that can be downloaded at https://vindr.ai/datasets/cxr. We released our mammogram-based Lesion Simulator and Lesion Remover at different training epochs (from 25, 50, 75, 100) to the public such that the interested researchers in the computer-aided detection field could utilize it for their work. They can be accessed at https://github.com/juhunlee82/Mammo-GAN-assisted-training. All codes for conducting this study are also available at the above link. The dataset will be made available to the scientific community on publication but should be reasonably requested from the corresponding authors. A signed data use agreement and institutional review board approval will be required before the release of research data.
